# P02.60. Characteristics of treatment adherence in low-income minority participants in a yoga dosing study for chronic low back pain

**DOI:** 10.1186/1472-6882-12-S1-P116

**Published:** 2012-06-12

**Authors:** A Boah, L Kwong, J Weinberg, K Sherman, R Saper

**Affiliations:** 1 Boston Medical Center, Boston, USA; 2 Boston University School of Public Health, Boston, USA; 3 Group Health Research Institute, Seattle, USA

## Purpose

Minority groups experience significant barriers to participation in clinical trials. Little is known about characteristics of treatment adherence among low income minority populations in CAM trials. This analysis aims to identify factors associated with class attendance, a major component of treatment adherence, in participants engaged in a research study of yoga for chronic low back pain (CLBP).

## Methods

We conducted the Yoga Dosing Study for 95 adults from Boston Medical Center (BMC) and five affiliated Community Health Centers (CHCs), all federally qualified and serving racially diverse low-income neighborhoods. The study consisted of a 12-week standardized hatha yoga protocol comparing once per week to twice per week 75-minute yoga classes. Activities to enhance adherence included: flexible yoga class schedules, weekly reminder phone calls, assistance with child care or transportation, make-up classes, attendance based raffles, staggered distribution of honoraria, and yoga take home practice supplies. We analyzed attendance rates at the end of the intervention for associations with demographic characteristics and pain related outcomes from data collected via questionnaires at baseline, 6, and 12 weeks. The Pearson Correlation and two sample t-tests were used for these analyses.

## Results

After 12 weeks, the mean attendance rates for the 1x/wk and 2x/wk groups were 73% and 62% respectively.(p=.11). Attendance rates were not associated with education, income, age, gender, race, change in low back pain score over 12 weeks, or length of time between enrollment into the study and beginning the intervention. Lower levels of low back pain intensity at 12 weeks was associated with higher attendance rates (p=.03).

## Conclusion

It is feasible to attain good treatment adherence for low income minority populations in a yoga research trial. Greater attendance rates were associated with lower levels of low back pain intensity at the end of the study, but not associated with demographics or treatment assignment.

